# 705. Burden of Medically-Attended Diarrhea and *Clostridioides difficile* Infection (CDI) Among Persons in the Community in an Integrated Healthcare Setting, 2016-2021

**DOI:** 10.1093/ofid/ofad500.767

**Published:** 2023-11-27

**Authors:** Sara Y Tartof, Mark A Schmidt, Richard Contreras, Fredrick J Angulo, Ana Florea, Joanna L Barreras, Judy L Donald, Joann M Zamparo, Deborah S Ling Grant, Elizabeth Shuster, Elisa Gonzalez, Jennifer L Kuntz

**Affiliations:** Kaiser Permanente Southern California, Pasadena, California; Center for Health Research, Kaiser Permanente Northwest, Portland, OR; Kaiser Permanente Southern California Department of Research & Evaluation, Pasadena, California; Pfizer, Inc., Portland, Oregon; Kaiser Permanente Southern California, Pasadena, California; Kaiser Permanente of Southern California, Pasadena, California; Kaiser Permanente Center for Health Research, Portland, Oregon; Pfizer Vaccines, Killingworth, Connecticut; Kaiser Permanente Southern California, Pasadena, California; Kaiser Permanente Center for Health Research, Portland, Oregon; Pfizer Vaccines, Killingworth, Connecticut; Kaiser Permanente Center for Health Research, Portland, Oregon

## Abstract

**Background:**

Population-based surveillance by the Centers for Disease Control and Prevention (CDC) indicates that the incidence of community-associated *Clostridioides difficile* infection (CDI) is increasing. However, CDI testing practices and incidence among patients seeking care for diarrhea in the outpatient setting have not been well described. Further, little is known about healthcare utilization and outcomes of patients with medically-attended CDI who are not subsequently hospitalized.

**Methods:**

We conducted a retrospective cohort study of Kaiser Permanente Southern California (KPSC) and Northwest (KPNW) adult (≥ 18 years of age) members from January 1, 2016 to December 31, 2021. We identified medically-attended diarrhea (MAD) in the outpatient setting through ICD-10 diagnosis codes. Among those with MAD, we identified *C. difficile* testing and outpatient CDI (oCDI), defined as a positive CDI test without hospitalization in the following 0-7 days. We described the demographic and clinical characteristics of patients with MAD and oCDI and calculated incidence rates (IR) of oCDI. We also described the occurrence of oCDI-associated healthcare visits, CDI testing, and oCDI treatment in the 12 months following oCDI diagnosis.

**Results:**

We identified 777,533 outpatient MAD episodes of which 93,964 (12.1%; 93,964/777,533) were tested for CDI. Among tested episodes, 10,110 (10.8%; 10,110/93,964)), involving 9,517 patients, had a positive CDI laboratory test. IR of outpatient oCDI decreased from 58.2 (95%CI: 55.7-60.7) per 100,000 person-years (PY) in 2016 to 45.7 (43.7-47.8) per 100,000 PY in 2021. Among 9,517 oCDI patients, 84.1% (n=8,006) CDI were ‘community-associated’ (without hospitalizations in the 84 days prior to index date) and only 44.1% (n=4,200) received an antibiotic in the previous 30 days. Only 6.7% (n=526) of oCDI patients had a CDI-associated hospitalization in the 12 months after diagnosis.

**Conclusion:**

There was a high incidence of MAD and CDI in among outpatients. The majority of our outpatient CDI population had no recent hospitalization and no recent antibiotic exposure. Further studies are needed to understand the factors associated with CDI diagnosed and managed in the outpatient setting.

**Disclosures:**

**Sara Y. Tartof, PhD MPH**, Genentech: Grant/Research Support|GSK: Grant/Research Support|Pfizer: Grant/Research Support|SPERO: Grant/Research Support **Mark A. Schmidt, PhD, MPH**, Intercept Pharmaceuticals: Grant/Research Support|Pfizer: Grant/Research Support|Vir Biotechnology: Grant/Research Support **Fredrick J. Angulo, DVM, PhD**, Pfizer Vaccines: Employee|Pfizer Vaccines: Employee|Pfizer Vaccines: Stocks/Bonds|Pfizer Vaccines: Stocks/Bonds **Ana Florea, PhD MPH**, Gilead: Grant/Research Support|GlaxoSmithKline: Grant/Research Support|Moderna: Grant/Research Support|Pfizer: Grant/Research Support **Joann M. Zamparo, MPH**, Pfizer Inc.: Full Time Employee|Pfizer Inc.: Stocks/Bonds **Deborah S. Ling Grant, PhD, MPH, MBA**, Pfizer: Grant/Research Support **Elisa Gonzalez, MPH**, Pfizer: Stocks/Bonds **Jennifer L. Kuntz, MS, PhD**, Pfizer: Grant/Research Support

